# Biased Allele Expression and Aggression in Hybrid Honeybees may be Influenced by Inappropriate Nuclear-Cytoplasmic Signaling

**DOI:** 10.3389/fgene.2015.00343

**Published:** 2015-12-01

**Authors:** Joshua D. Gibson, Miguel E. Arechavaleta-Velasco, Jennifer M. Tsuruda, Greg J. Hunt

**Affiliations:** ^1^Department of Entomology, Purdue University, West LafayetteIN, USA; ^2^CENID-Fisiología y Mejoramiento Animal, Instituto Nacional de Investigaciones Forestales, Agrícolas y PecuariasMéxico, Mexico; ^3^Public Service and Agriculture, Clemson UniversityClemson, SC, USA

**Keywords:** parental effects, cytoplasmic incompatibility, hybrid incompatibility, aggression, Africanized, *Apis mellifera*, PIWI, PIWI-interacting small RNAs

## Abstract

Hybrid effects are often exhibited asymmetrically between reciprocal families. One way this could happen is if silencing of one parent’s allele occurs in one lineage but not the other, which could affect the phenotypes of the hybrids asymmetrically by silencing that allele in only one of the hybrid families. We have previously tested for allele-specific expression biases in hybrids of European and Africanized honeybees and we found that there was an asymmetric overabundance of genes showing a maternal bias in the family with a European mother. Here, we further analyze allelic bias in these hybrids to ascertain whether they may underlie previously described asymmetries in metabolism and aggression in similar hybrid families and we speculate on what mechanisms may produce this biased allele usage. We find that there are over 500 genes that have some form of biased allele usage and over 200 of these are biased toward the maternal allele but only in the family with European maternity, mirroring the pattern observed for aggression and metabolic rate. This asymmetrically biased set is enriched for genes in loci associated with aggressive behavior and also for mitochondrial-localizing proteins. It contains many genes that play important roles in metabolic regulation. Moreover we find genes relating to the piwi-interacting RNA (piRNA) pathway, which is involved in chromatin modifications and epigenetic regulation and may help explain the mechanism underlying this asymmetric allele use. Based on these findings and previous work investigating aggression and metabolism in bees, we propose a novel hypothesis; that the asymmetric pattern of biased allele usage in these hybrids is a result of inappropriate use of piRNA-mediated nuclear-cytoplasmic signaling that is normally used to modulate aggression in honeybees. This is the first report of widespread asymmetric effects on allelic expression in hybrids and may represent a novel mechanism for gene regulation.

## Introduction

The honeybee (*Apis mellifera*) is becoming a promising model for understanding epigenetic processes, which affect gene expression without modifying the DNA sequence. Honeybees possess all of the key genes in the methylation machinery (Wang et al., 2006) and DNA methylation plays a role in queen caste determination (Kucharski et al., 2008). Additionally, honeybee post-translational histone protein modifications have been characterized and may play a role in caste determination (Spannhoff et al., 2011; Dickman et al., 2013). However, we are just beginning to learn how these epigenetic processes regulate gene expression in honeybees. For example, methylation events in the bee genome have been shown to be plastic and related to behavioral castes (Herb et al., 2012), though they appear to be associated with alternative splicing rather than large alterations in transcriptional abundance (Flores et al., 2012; Foret et al., 2012).

One major hypothesis for epigenetic control of gene expression in honeybees, the kinship theory of genomic imprinting, predicts a bias in the expression of alleles in a parent-specific manner due to differences in relatedness between nestmates in order to enhance inclusive fitness (Haig, 2002; Queller, 2003). Another hypothesis predicts a bias in expression toward the maternal allele in order to maintain a match between co-adapted nuclear alleles and the maternally inherited mitochondria (Wolf, 2009). There is considerable empirical support for the former theory while there is little evidence in support of the latter (Haig, 2004). Only the kinship theory predicts a paternal expression bias, but both theories predict a maternal bias for some genes and in these cases support for one theory over the other can only be distinguished by the functions of the biased genes. In either case, this differential allelic expression must include an epigenetic mechanism because the expression bias of an allele is affected by the parent from which it is inherited and not solely by its genotype (i.e., European or Africanized alleles). Until recently, it was not known if there were gene expression effects in honeybees that were consistent with epigenetic regulation. However, there are phenotypic parental effects on aggression and metabolic rate that have been identified in studies utilizing hybrids from crosses between European and African subspecies that occur in patterns that suggest that epigenetic processes may be involved (Harrison and Hall, 1993; Guzman-Novoa et al., 2005; Oldroyd et al., 2014).

We previously tested for epigenetic effects by identifying parent-specific gene expression (PSGE) in honeybees by utilizing reciprocal F1 worker families derived from crosses of European (*A.m. carnica*) and Africanized bees (invasive hybrids between African *A.m. scutellata* and European honeybees; Kocher et al., 2015). Transcriptomes of workers in the two reciprocal families (differing in the lineage from which each parent is derived) were sequenced and read counts of heterozygous SNPs were used to test for PSGE. This experimental design allows us to assess epigenetic effects on transcription because the genotype (European or Africanized) and parent-of-origin of an allele will differ between the two families. Each allele will be inherited maternally in one family and paternally in the other. We found that PSGE is present in the honeybee (1–2% of tested loci) and that a bias toward maternal expression was common. A set of 46 genes showed consistent, symmetric parental biases in both families (maternal or paternal in both families) and several of these genes have functions that are predicted by the kinship theory of genomic imprinting. Surprisingly, a strong maternal bias occurred primarily in the family with European maternity (EA hybrids hereafter) and 215 genes were maternally biased exclusively in this family compared to only 24 genes that were maternally biased exclusively in the Africanized maternity family (AE hybrids hereafter). This was the first evidence of PSGE in honeybees and, while PSGE has been studied in many organisms, to the best of our knowledge the observed asymmetric pattern of PSGE (PSGE in only one family) has not been documented in other species.

Asymmetries in phenotypic effects between reciprocal hybrid families are commonly observed, including honeybee hybrids (Harrison and Hall, 1993; Guzman-Novoa et al., 2005; Turelli and Moyle, 2007). These asymmetric phenotypic effects require that there be some asymmetry in the expression of genes inherited from the parents, which could include sex chromosomes, cytoplasmic factors, differentially imprinted genes, or maternal effects (Wolf et al., 2014). We propose that these asymmetric phenotypic effects in honeybees are due to asymmetric PSGE that is the result of inappropriate signaling in the hybrids. Specifically, we propose that these wide crosses disrupt, in the hybrids, nuclear-cytoplasmic signaling pathways, and epigenetic processes that are utilized differentially in the parental lineages. This disruption leads to inappropriate signaling in these pathways that influences epigenetic chromosomal modifications (in an allele-specific manner), ultimately resulting in asymmetric phenotypic effects. In support of this, some of the maternally biased genes in our previous study were located within quantitative trait loci (QTL) that influence honeybee stinging behavior, a trait which is asymmetrically expressed in these hybrids (Hunt et al., 1998, 2007; Guzman-Novoa et al., 2005). In addition, the maternally biased genes for one of the three tissue samples analyzed (first instar larvae) were enriched for nuclear-encoded proteins known to localize to the mitochondria, supporting a connection between the cytoplasm, asymmetric PSGE, and asymmetric hybrid effects. Here we undertake a more comprehensive analysis of the transcriptome data to identify additional genes that show bias in these hybrids, to characterize their function and chromosomal localization with respect to QTL, and to test for differential gene expression between the two families.

## Materials and Methods

### Previous and New Analyses

The work presented here utilizes a dataset originally published in Kocher et al. (2015). The sequencing data are available in the NCBI Short Read Archive, project number PRJNA277772. This dataset consisted of the cross utilizing Africanized honeybees (AHB) and the European honeybee (EHB) subspecies *A.m. carnica* (described in “crosses” below) and the analyses leading to the expression levels of the alleles in the reciprocal hybrid families (described in “PSGE of alleles” below). We produced a new gene set by altering the criteria for a gene to be considered to show PSGE and differences in these criteria are described below. New analyses were performed using this gene set, constituting the results presented here. The new work also includes new crosses utilizing the European subspecies *A.m. ligustica* and *A. m. carnica* that provided workers used in the stinging behavior assays.

### Crosses

Crosses for evaluating PSGE were previously described in Kocher et al. (2015) and biased expression patterns were further analyzed from this dataset. Briefly, EHB and AHB colonies were maintained at INIFAP facilities near Villa Guerrero, Estado de México, Mexico. There are several closely related subspecies of EHB that are often used by beekeepers and two of these were among the colonies tested for use in our crosses, Carniolan bees, *A.m. carnica*, and Italian bees, *A.m. ligustica*. The two most aggressive AHB colonies and the most docile EHB colonies (one each of *A.m. carnica* and *A.m. ligustica*) were chosen based on differences in stinging behavior and response to queen mandibular pheromone. Daughter queens and drones were raised from the parental colonies. Pairs of reciprocal crosses were performed using single-drone queen instrumental insemination between one AHB and the *A.m. ligustica* parental colony and between the AHB and the *A.m. carnica* parental colony. For crosses with *A. m. carnica*, two EA families and four AE families were tested for individual stinging behavior (see below). For crosses with *A. m. ligustica*, three EA and three AE families were tested.

### PSGE of Alleles

**FIGURE 1:**
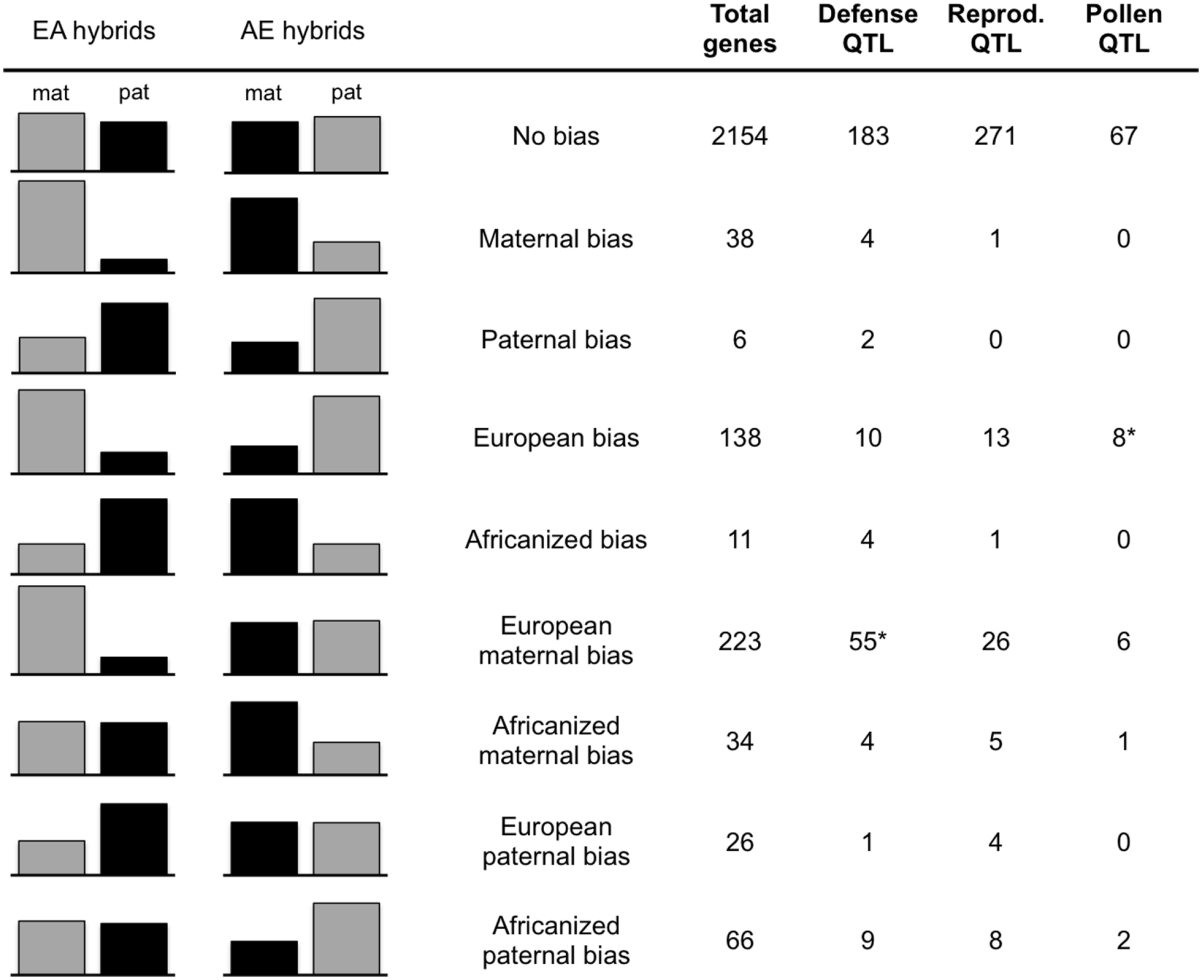
**Gene counts in bias categories**. Average maternal/paternal bias of all genes in each bias category in each hybrid family (EA, European maternity; AE, Africanized maternity). Gray columns = European allele, Black = Africanized allele. The total number of genes in each category and the number falling in each QTL type are given in the columns on the right. Only genes falling into a single bias category across samples are included in counts for QTL types. ^∗^Significantly more genes in this category are present within these QTL than expected by chance (Bonferroni corrected *p*-value = 0.005).

Expression levels of the alleles in the two reciprocal crosses are from Kocher et al. (2015). Briefly, all transcriptome data is derived from the two reciprocal crosses utilizing the *A.m. carnica* queen (designated EA and AE for those with EHB and AHB maternity, respectively). Transcriptomes were sequenced from cDNA libraries of pooled first instar larvae (two libraries per family), pooled adults (guard bees, two libraries per family), and individual adult brains (three libraries per family). Single nucleotide polymorphisms (SNPs) differentiating European and Africanized alleles were identified by sequencing genomic DNA of the queen and drone parents of these two crosses to ensure the European and Africanized alleles were homozygous and different, resulting in F1 offspring that are heterozygous at all tested SNPs. All reads were mapped to the honeybee reference genome (Amel4.0; The Honeybee Genome Sequencing Consortium, 2006). Using counts of reads at each SNP, a general linear interactive mixed model (SAS, Cary, NC, USA) was utilized to assess expression of each allele for all transcripts containing diagnostic SNPs. The analysis in Kocher et al. (2015) required the bias to be in the same direction (maternal or paternal) in both directions of the cross (EA and AE hybrids) based on significant parent FDR < 0.05, and a bias of at least 0.6 (maternal or paternal reads/total reads; Wang and Clark, 2014) in order to search for consistent parent-of-origin effects. For the current analysis, we relaxed this criteria and only required that the bias be present in one direction of the cross. These genes were then placed into bias categories based on the expression levels of their alleles in each family relative to the parent-of-origin of that allele (e.g., Maternal bias, EA maternal/AE maternal; European bias, EA maternal/AE paternal, etc.; see **Figure 1**).

### Individual Stinging Behavior

**FIGURE 2:**
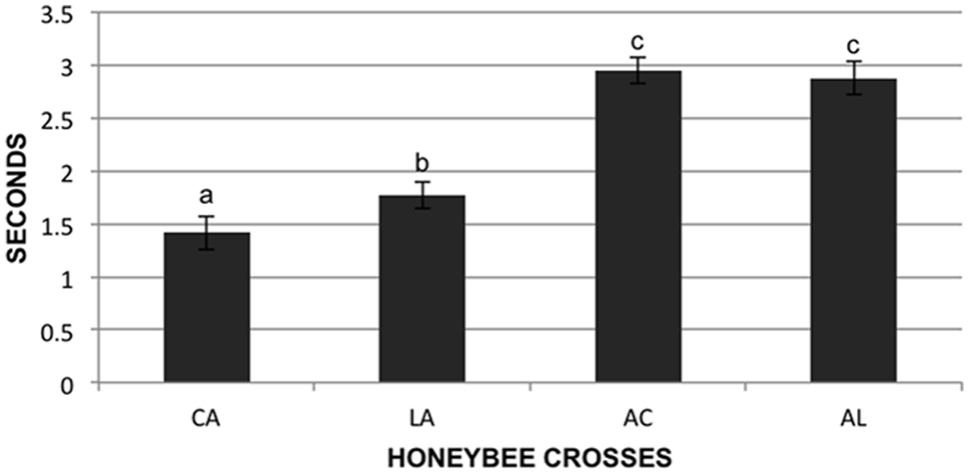
**Sting response time of individuals**. Individual bees (573 total) were given an electrical shock from a constant current stimulator and the time in seconds for them to sting a suede patch was recorded. Genotypes of reciprocal hybrids are given on the X-axis: Africanized maternity hybrids AC (*Apis mellifera carnica* father) and AL (*A.m. ligustica* father), and Africanized paternity hybrids CA (*A.m. carnica* mother) and LA (*A.m. ligustica* mother). Data presented is untransformed, letters designate significant differences.

We tested the stinging behavior of individual bees from our crosses by measuring the time that each bee took to sting a black suede patch after being stimulated with electrical current (assay described in Shorter et al., 2012). In total 573 bees were tested from the four F1 reciprocal colonies. These colonies are designated AL (AHB queen × *A.m. ligustica* drone), LA (*A.m. ligustica* queen × AHB drone), AC (AHB queen × *A.m. carnica* drone), and CA (*A.m. carnica* queen × AHB drone) in **Figure 2**. Data was transformed using the natural log function to fit a normal distribution and was analyzed under a one way analysis of variance to test for differences in the stinging behavior of the four F1 reciprocal crosses. Least squares means *t*-tests were performed to compare the means of the four groups.

### Differential Expression Analysis

We assessed differential gene expression (DGE) between comparable stages/tissues in the EA and AE families using CLC Genomics Workbench version 7.5 (CLC Bio, Boston MA, USA) employing the Empirical Analysis of DGE option, which implements the “Exact Test” of Robinson et al. (2010). Genes were considered significantly differentially expressed if the False Discovery Rate corrected *p*-value was less than 0.05.

### Overlap of Biased Genes with Known QTL

We assessed whether genes showing asymmetric maternal PSGE lie within QTL influencing traits related to colony defense (Hunt et al., 1998, 1999, 2007; Arechavaleta-Velasco et al., 2003; Shorter et al., 2012), reproduction (Oxley et al., 2008; Linksvayer et al., 2009; Rueppell et al., 2011), and pollen foraging behavior (Hunt et al., 1995, 2007; Page et al., 2000). We used the diagnostic SNPs within biased genes to determine their location in the Amel4.5 assembly. In cases where physical locations of markers were given in the QTL studies, we used these to identify the bounds of the QTL (typically the 1.5 LOD support interval). When information on physical locations of markers wasn’t available, we used the sets of candidate genes from these projects to identify the range of the QTL. We then compared the positions of the biased genes with the ranges of these QTL to determine overlap.

### Genomic Clustering of Biased Genes

In addition to identifying biased genes that are within previously identified QTL, we also assessed whether any biased genes were in physical clusters within the genome by visualizing their positions on SNP-based linkage maps (Arechavaleta-Velasco et al., 2012; Tsuruda et al., 2012). Once putative clusters of significantly biased genes were identified, we also looked at the allelic expression patterns of all tested genes (regardless of significance) within the putative cluster.

### Statistical Tests of Enrichment/Overlap

We utilized goodness of fit tests to determine whether the genes in our bias categories (see **Figure 1**) were enriched for mitochondrial-localizing genes, significantly overlapped other gene sets, or were overrepresented in QTL. In all cases we only report results if they were significant after Bonferroni correction. We tested for enrichment of mitochondrial-localizing proteins by performing reciprocal BLASTs of the AmelOGS3.2 peptide sequences against a set of *Drosophila melanogaster* genes with proteins that are known to localize to mitochondria (Pagliarini et al., 2008; Smith et al., 2012). Expected values for our biased gene categories were calculated using the proportion of genes in the total honeybee gene set that match the *Drosophila* mitochondrial-localizing set. We also tested for significant overlap of our genes in each bias category with our own differentially expressed gene set, genes differentially expressed between aggressive and non-aggressive bees (Alaux et al., 2009), and for overrepresentation within QTL. We used the proportion of the total official gene set represented in each of these groups to calculate the expected number of genes in each of our bias categories. We also tested for Gene Ontology (GO) term enrichment using the best reciprocal matching *D. melanogaster* genes by utilizing the Gene Ontology Consortium’s enrichment analysis pipeline (geneontology.org).

### Use of Animals in Research

This research did not require IRB approval because we only used invertebrates in this study, which are exempt from IRB approval. Despite not requiring approval, we made every effort to minimize any potential suffering of the bees used in this research.

## Results

### Genes Showing PSGE Bias

We found that out of the 2663 unique transcripts that we could test, 509 exhibited biased expression with one of the parental alleles used more than the other (≥0.6 bias) in at least one reciprocal hybrid family. In addition to the previously reported genes that show a parent-of-origin effect (either maternal or paternal in both families), we found evidence for biased PSGE in all other potential categories of bias (maternal or paternal only, allele-specific, or no bias in either family; **Figure 1**). Over 40% (223 genes) of the biased genes had a maternal bias only in the hybrids with European maternity (EA hybrids; **Figure 1**). Out of these 509 transcripts, 33 fell into more than one category of bias due to differences between samples (Supplementary Tables S1 and S2). In the majority of these cases, the category shift was due to a small level of bias in the AE family (near the 60% cutoff value) relative to the greater bias in the EA family. This is evident because the number of transcripts falling into more than one category decreases by 85% (to 5 total) simply by increasing the cutoff value to 70% bias. The EA maternal bias is much more robust, as indicated by the decrease of only <2% in the EA maternal-only category with the same change in criteria (Supplementary Table S2 and Figure S1). Nevertheless, to ensure unambiguous results we removed these genes for tests of enrichment of mitochondrial-localizing genes and presence in QTLs.

### Differential Expression Analysis

A total of 160 unique genes were differentially expressed between the EA and AE families in at least one stage and six of these genes were differentially expressed in both guards and another stage while only one gene was shared between larvae and brains (Supplementary Table S3). One-hundred and one of these genes have a more than twofold change in expression.

### Parental Effect on Stinging

There were significant differences in the honeybee individual stinging behavior between the four F1 reciprocal crosses (*F* = 53.64; df = 3,567; *p* < 0.01). The bees of the colonies with European maternity (CA and LA) stung significantly faster than the bees of the colonies with Africanized mothers (AC and AL; *p* < 0.05). There were differences between the two crosses with European maternity, the bees with *A.m. carnica* maternity (CA) stung faster than the bees of the cross with *A.m. ligustica* maternity (LA; *p* < 0.05), but there were no differences in the time to sting for the two crosses with Africanized maternity (AC and AL; **Figure 2**; *p* > 0.05).

### Genomic Clustering of Biased Genes

**FIGURE 3:**
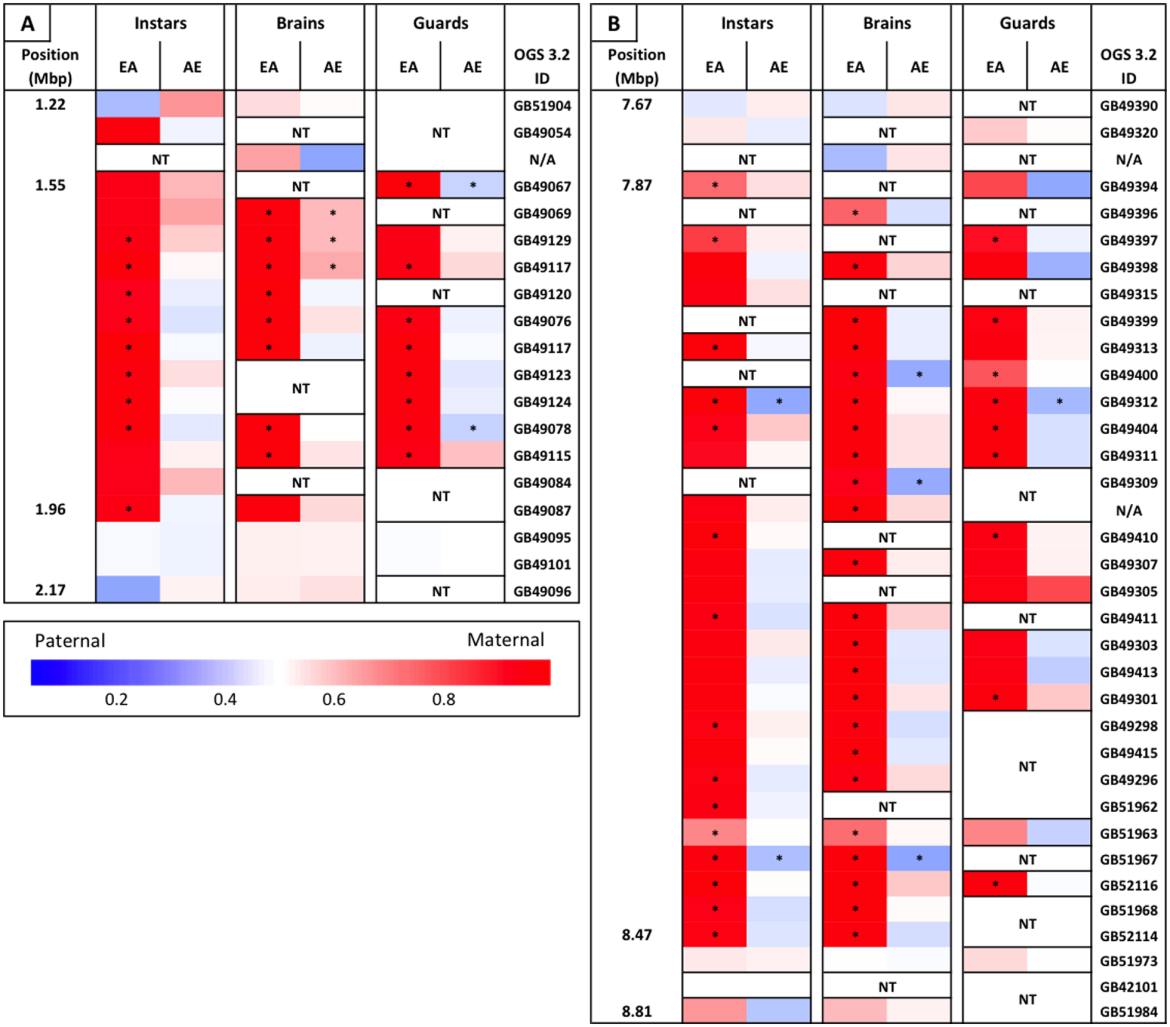
**Heat map of maternally biased gene clusters. (A)** Gene cluster on chromosome 3, overlapping a QTL associated with stinging behavior. **(B)** Gene cluster on chromosome 12, overlapping a QTL associated with alarm pheromone production (isopentyl acetate). Position along the chromosome (in Mbp), relative allele usage of each reciprocal family within each sample, and OGS 3.2 gene ID is given for every tested gene within these clusters. Relative allele usage calculated as maternal read count/total read count. ^∗^Statistically significant allelic bias. NT, not tested. N/A in gene ID column are transcripts that had no clear match to a protein coding gene.

We identified two regions containing clusters of genes that all appear to be biased, both of which overlap with defense-related QTL. One on chromosome 3 lies within the Sting-2 QTL, a region associated with increased colony-level stinging behavior (Hunt et al., 1998, 2007). There are 12 genes within a region of ∼410 kb that show a significant maternal bias of greater than 90% in the European maternity family (**Figure 3A**). There is only one additional gene within this region that could be tested and this gene also shows >90% maternal bias in this family. The second cluster lies on chromosome 12 within the bounds of a QTL associated with production of the active component of alarm pheromone, isopentyl acetate (Hunt et al., 1999). This region is ∼600 kb in length and there are 29 genes that could be tested within this region. Similar to the cluster within the Sting-2 QTL, 27 of these genes show a significant maternal bias (>90% maternal in 23 of these genes) in the European maternity family and the remaining two genes show the same pattern of extreme maternal bias (**Figure 3B**).

### Overlap of Biased Genes with Other Data Sets

We found 164 of our biased genes overlapping with known QTL associated with traits for defense, reproduction and foraging behavior based on the position of these genes in OGS3.2 (Elsik et al., 2014). Within these QTL, just two of the gene bias categories were over-represented relative to the expected number based on OGS3.2 (**Figure 1**). The European maternal biased (only maternal bias in EA) gene set is overrepresented in defensive QTL with 55 genes (expect 36.3, *p* < 0.002), and the EA maternal AE paternal (European biased) gene set is overrepresented in pollen hoarding QTL with eight genes (expect 2.9, *p* < 0.0031).

We tested whether any of our biased categories are enriched for genes whose proteins are known to localize to mitochondria and found that the genes that are maternally biased only in the EA family are significantly enriched in each of the three samples (larvae [10/78], *p* = 0.0005; brains [16/140], *p* = 0.0001, adults [7/49] *p* = 0.0016). Moreover, 15 of the 17 genes that have the same bias in all three sample types are maternally biased only in the EA family and are highly enriched for mitochondrial-localizing genes (6/15, *p* = 9.4 × 10^-11^). Despite genomic clustering of some of our biased genes, there is no clustering of mitochondrial-localizing genes. We also determined the extent to which our gene list overlapped with genes that were differentially regulated in aggressive vs. non-aggressive bees (Alaux et al., 2009). Significantly more genes overlapped between this study and our biased gene list than expected by chance (115 genes, *p* = 0.017), though this is not significant if we correct for testing each individual bias category. None of the individual categories are significantly enriched for overlapping genes, even without multiple test correction (Supplementary Table S1). Similarly, 43 of our differentially expressed genes overlap with those of Alaux et al. (2009), though there is no pattern to the overlap in regards to the up or down regulation of the genes in each study (Supplementary Table S3).

## Discussion

We previously found evidence of PSGE in honeybees (Kocher et al., 2015). However, we also found over 200 genes that showed highly biased expression toward the maternal allele, but only in the family with European maternity (EA hybrids). This asymmetric bias in expression is not predicted by theories of genomic imprinting. Similarly, if the pattern we observed were due to allelic effects (Africanized or European alleles preferentially expressed), we would expect to see a maternal bias in one family and a paternal bias in the other, but we did not. There have been several cases reported in which asymmetric hybrid phenotypic effects have resulted from disrupted genomic imprinting in hybrids. One example is found in two species of deer mice, *Peromyscus maniculatis* and *P. polionotus*, in which the hybrid family with a *P. polionotus* mother exhibits offspring overgrowth while the reciprocal family exhibits undersized offspring (Loschiavo et al., 2007; Duselis and Vrana, 2010; reviewed in Wolf et al., 2014). These asymmetric hybrid effects are expected because of the imprinting that is predicted to occur in *P. maniculatis*, but not in *P. polionotus*, based on the conflict hypothesis of imprinting (Moore and Haig, 1991). *P. maniculatis* is polygamous while *P. polionotus* is monogamous, and therefore there is selection for *P. maniculatis* fathers to increase their own offspring’s growth at the expense of other male’s offspring from the same mother. This inclusive fitness incentive doesn’t exist in a monogamous system. In *P. maniculatis* there is also a selective advantage for the mother to counteract this with mechanisms that allow all of her offspring to receive equal nutrition. The hybrid families end up with asymmetric offspring growth because the genomic conflict (and hence the balanced offspring growth) is disrupted in these crosses. *P. polionotus* parents don’t have this conflict and so don’t counteract the growth effects of their *P. maniculatis* partners, resulting in small offspring with *P. maniculatis* mothers and large offspring with *P. maniculatis* fathers. We extend this idea to hybrids between races of honeybees by proposing that the disruption of these parental effects (whether imprinting or other heritable factors) results in inappropriate signaling within established nuclear-cytoplasmic signaling pathways that leads to allele-specific changes in expression in only one of the hybrid families. To the best of our knowledge, there are no other examples of PSGE biases that show a widespread asymmetric pattern such as we find in these reciprocal hybrids.

Our biased gene set is dominated by a single category of bias, genes that are maternally biased only in the European maternity family (EA hybrids; **Figure 1**). The second most abundant category is bias toward European alleles, which may be influenced by similar processes. Previous studies investigating several trait differences between AHB and EHB found phenotypic patterns in hybrid crosses that are similar to the asymmetry we see in allelic bias. EA bees repeatedly exhibit high Africanized-like aggression while AE hybrids exhibit levels of aggression intermediate to the parents (Guzman-Novoa et al., 2005). We tested whether our biased genes may play a role in this asymmetric aggression by assessing the positions of the biased genes in the Amel4.5 assembly to see how they may fit with previously identified QTL associated with aggressive behavior, as well as QTL associated with reproduction and foraging (Elsik et al., 2014). We found that 89 out of the total of 509 biased genes lie within QTL for defensive traits, 58 genes are within QTL for reproductive traits and 17 are within QTL for pollen foraging behavior (**Figure 1**). There were significantly more genes than expected by chance between the EA maternal-only bias category and defensive QTL (*p* = 0.0029) and between the overall European bias category (EA maternal and AE paternal) and pollen foraging QTL (*p* = 3.39 × 10^-5^). The connection between the European bias group and pollen foraging is interesting given that the propensity for pollen collection has been shown to vary between European and Africanized bees (Pesante et al., 1987; Page et al., 2000). However, unlike the genes with an EA maternal-only bias, when we increase our bias cutoff criteria from 60 to 70%, the number of genes in the pollen foraging QTL category is reduced by more than 60% and the enrichment within these QTL disappears, indicating that these genes are not highly biased in either family (Supplementary Table S2). We also tested whether our biased gene set is enriched for genes that are differentially expressed between aggressive and non-aggressive bees (Alaux et al., 2009). While the entire set of biased genes shows a slight enrichment for these genes (115 biased genes overlapping with 2254 differentially expressed genes; *p* = 0.017), no individual category of bias is significantly enriched.

Another asymmetric phenotype is that EA hybrids have asymmetrically low flight metabolic capacity (based on whole body CO_2_ measurements) relative to both the parents and AE hybrids, which could indicate that the there is an incompatibility between maternally derived European mitochondria and paternally derived nuclear genes in EA hybrids (Harrison and Hall, 1993). A separate study of aggression in honeybees found that aggression and brain metabolic rates are related, as the brains of highly aggressive bees showed significantly reduced oxidative metabolism relative to non-aggressive bees (Alaux et al., 2009). Reducing the rate of oxidative phosphorylation both in bees and in *Drosophila*, even in the whole body, increased aggression and therefore it appears that brain metabolic rate plays a causal role in aggressive behavior in insects (Li-Byarlay et al., 2014). Given this connection, differential gene expression associated with aggression in the parents (Alaux et al., 2009) would lead us to expect to find that metabolic genes show differential expression between our reciprocal families. Despite this expectation, genes that were differentially expressed between these families are not enriched for any functional GO category or for mitochondrial-localizing proteins (Supplementary Table S3; See Supplementary File 1 for discussion of differentially expressed genes). The lack of enrichment for genes showing expression differences between aggressive and non-aggressive bees or for any functional GO categories makes interpretation of our differentially expressed gene set difficult. Interestingly, only 13 of the 509 biased genes are also differentially expressed between the two families. This is significantly more overlap than expected given the small number of differentially expressed genes (*p* = 0.0008, Supplementary Table S1), however, there was no pattern to the overlap between up/down regulation and bias category. The fact that the vast majority of the biased genes are not differentially expressed means that in general there is a combination of allele-specific silencing and dosage compensation and that this process is occurring for many genes in only one of the reciprocal families. These results are reminiscent of the increased expression (e.g., *Drosophila* males) or silencing (e.g., mammalian females) that occurs on sex chromosomes to maintain comparable expression in both sexes (Disteche, 2012).

If nuclear-mitochondrial interactions are involved in the asymmetric phenotype of EA aggressive behavior through changes in metabolism, then we expect that this phenotype would have an inherent physiological basis and not necessarily be influenced by social interactions. Therefore we used an aversive stimulus of individuals in a lab assay to test for an asymmetric phenotype outside the colony environment. We used hybrids with two European mitochondrial backgrounds (*A.m. ligustica* and *carnica*) for these tests. As seen at the colony level, bees with European mothers reacted more aggressively (faster to sting) than those with Africanized mothers but also that bees with *A.m. carnica* mothers were significantly more aggressive than those with *A.m. ligustica* mothers (**Figure 2**) even though the *A.m. carnica* parental source was less aggressive than the *A.m. ligustica* parent (data not shown). The stinging behavior QTL discussed above were also identified in a cross with *A.m. carnica* mothers (Hunt et al., 1998).

In addition to the significant overlap with QTL associated with aggressive behavior, another clue that the asymmetric PSGE in the EA family may be tied to both the asymmetric hybrid aggression and metabolic deficit is the fact that we found highly significant enrichment for mitochondrial-localizing proteins in every sample (Supplementary Table S1). This contrasts with results of our previous analyses that focused on 46 genes that showed consistent parental effects in both families. That study only found significant enrichment of mitochondrial proteins for biased genes in larvae of the EA family (Kocher et al., 2015). It is important to note that if the asymmetric bias in EA hybrids were due solely to incompatible interactions between nuclear genes and their proteins that directly interact with mitochondria, we would expect this enrichment to be very high (approaching 100%). However, enrichment only reaches ∼8% in this biased gene set, which implies that this asymmetric bias may be due to dysfunctional signaling involving the mitochondrial and nuclear genomes rather than a direct result of nuclear-mitochondrial dysfunction. The reduced oxidative brain metabolism associated with aggression in bees isn’t necessarily an overall reduction in energy metabolism, as studies show this reduction in oxidative metabolism is accompanied by a shift toward aerobic glycolysis (AG; glycolysis in the presence of oxygen; Chandrasekaran et al., 2015). The shift toward AG is mediated by mitochondrial retrograde signaling (signals from mitochondria that regulate nuclear transcription), a process that is normally used to maintain energy homeostasis (Liu and Butow, 2006). These connections may implicate retrograde signaling in the modulation of aggression in bees (Li-Byarlay et al., 2014).

In addition to this newfound connection with aggression in bees, the shift away from oxidative metabolism and toward AG is a well established phenomenon in cancer cells, known as the Warburg effect (Warburg, 1956). This metabolic shift is thought to play an important role in cell proliferation in both cancer cells and in normal, non-cancer cells (Lunt and Vander Heiden, 2011). AG seems to be especially important in brain tissue, as developing brain tissue shows this same metabolic transition and brain areas with increased synaptic activity exhibit major changes in lactate concentrations, a byproduct of AG (Barros, 2013; Gershon et al., 2013; Goyal et al., 2014). GO analysis of our EA maternal-only biased gene set revealed an enrichment for 80 GO terms but these fall into a few broad categories that include cellular morphogenesis (particularly neurogenesis), behavior, and regulation and cell signaling (Supplementary Table S4). These categories are consistent with both the behavioral changes in the EA family as well as the cellular processes associated with a metabolic switch toward AG. Although genes that were maternally biased in EA were enriched for mitochondrial-localizing proteins, this set is not enriched for any GO categories directly involved in energy metabolism. However, the biased gene set does contain many genes that likely play a role in the retrograde response that elicits the switch toward AG. Moreover, this gene set has many genes that play a role in transcriptional regulation, including the piwi-interacting RNA (piRNA) pathway, which is a small RNA pathway involved in chromatin modifications (Huang et al., 2013). While we couldn’t test for these small RNAs due to the size selection involved in our library preparation, these genes may provide a link between the metabolic shift to AG involving mitochondrial signaling and a potential mechanism for the asymmetric maternal-only bias that we see in gene expression (see Supplementary File 1 for a discussion of our interpretation of the connection these genes provide).

The piRNA pathway acts by modifying chromatin to inhibit transcription, as compared to posttranscriptional silencing initiated by other small RNA pathways (Huang et al., 2013). The piRNA pathway is primarily involved in the suppression of transposable elements, but recent studies have shown that piRNAs also play important roles in epigenetic modulation and genomic imprinting (Brennecke et al., 2008; Chang et al., 2009; Huang et al., 2013; Le Thomas et al., 2013). Chromatin modifications may help to explain our lack of differential expression of biased genes between the hybrid families: if the paternal allele is unable to be expressed due to these modifications, then any signal in the cell to increase expression (e.g., transcription factor binding) will only be able to act on the maternal allele, resulting in both allelic bias and a lack of differential expression. Chromatin modifications may also explain why two of the defensive QTL identified above contained large clusters of significantly biased genes in which every gene that could be tested showed the same pattern of >90% maternal bias only in the EA family (**Figure 3**). In both of these QTL we were only able to test a subset of all the genes in the region due to non-informative SNPs and/or insufficient read counts (Sting 2, 14/57; alarm pheromone, 27/59), however, the consistent level of bias in tested genes and their broad distribution across these clusters indicates that this bias likely occurs across all genes within these clusters. Within these clusters the same genes are biased across all sample types (though not always significant due to read counts), which indicates that this pattern is likely present throughout the lifespan of the individuals and across all tissues. It is possible that there is an inversion in these regions in the AHB lineage resulting in this pattern of expression bias, but we are unable to test for this due to low coverage of our genomic DNA from this lineage. This possibility seems unlikely, however, as we would expect to see a comparable reduction in Africanized (i.e., maternal) expression in the AE family in these regions and previous independent studies of recombination in EA hybrids haven’t indicated the expected loss of recombination within these regions (Hunt et al., 1998; Shorter et al., 2012; Ross et al., 2015). Given the size of these clusters (∼500 kb) and the near complete silencing of the paternal alleles across tissues and life stages it seems likely that differential chromatin modifications in the homologous chromosomes contribute to this asymmetric pattern. While it is possible that these chromatin modifications occur independent of the piRNA pathway (e.g., chromosomal conformational changes that can be assessed using the Hi-C technique; Belton et al., 2012), these also seem less likely to be responsible for the overall asymmetric bias than the piRNA pathway due the genes involved in this bias (see Supplementary File 1) and the fact that >80% of these asymmetrically biased genes lie outside of these clusters (223 genes total with 39 in clusters). Given that the piRNA pathway acts in a sequence specific manner and may therefore be able to act on individual genes (Huang et al., 2013), we consider these other possibilities as alternative hypotheses to the model we propose below.

Piwi-interacting RNAs are loaded into oocytes, where they serve as a sequence-specific transgenerational epigenetic memory of both gene silencing and activation (maternal licensing), and in self/non-self recognition. piRNAs have also recently been found in mature sperm (Johnson and Spence, 2011; Shirayama et al., 2012; Pantano et al., 2015). The piRNA pathway has been shown to be involved in epigenetic regulation of phenotypic traits in both mice (white tail tip, WTT; Yuan et al., 2015) and fruit flies (ectopic long bristle outgrowths on the eyes, ELBOs; Sollars et al., 2003; Gangaraju et al., 2011). Both of these phenotypes occur at a low frequency in populations (naturally for WTT mice and artificially induced for ELBOs in *Drosophila*) and represent an epigenetic capacity within these populations that is normally suppressed through the piwi/piRNA pathway. The epigenetic capacity for these phenotypes can be released through selection for these traits (Sollars et al., 2003; Ruden et al., 2015; Yuan et al., 2015). These phenotypes are initially expressed in individuals with certain mutant alleles but the phenotype can occur in offspring that lack the causal mutant (Ruden et al., 2015; Yuan et al., 2015). Perhaps most intriguingly, the ELBOs in *Drosophila* can be maintained in the population (over 100 generations) as long as selection for the trait is maintained (Ruden et al., 2008). This epigenetic selection may help to explain our asymmetric hybrid effects.

**FIGURE 4:**
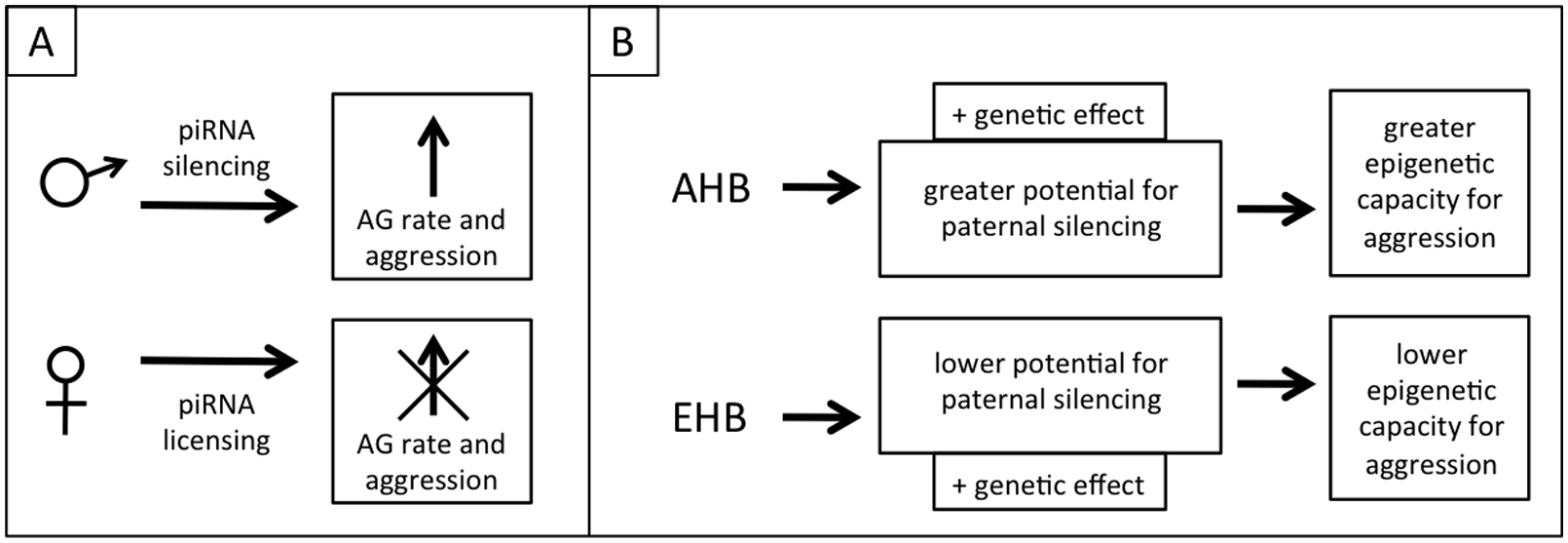
**Proposed model of epigenetic regulation of aggression through piRNAs. (A)** Divergent selective pressure for aggressive reproductive offspring on males and females creates genomic conflict, but this conflict is balanced by the need for appropriate colony-level aggression. Fathers attempt to increase aggression (through a shift toward aerobic glycolysis, AG) by silencing genes using sperm-loaded piRNAs. Mothers negate this silencing through genomic licensing using oocyte-loaded piRNAs. **(B)** AHB and EHB differ in aggression due to both genetic effects and a greater epigenetic potential for aggression in AHB, selected for in either their native or introduced range.

We speculate that the metabolic switch toward AG in honeybee brains and the associated aggression is a phenotypic trait that has a partially epigenetic basis, mediated through the piwi/piRNA pathway. An epigenetic switch to aggression is at least implied by theory regarding genomic imprinting in honeybees, as honeybees exhibit extreme polyandry and drones should have an evolutionary drive to produce daughters that are more selfish in regards to producing their own offspring, including more aggressive daughter queens that may be successful in queen duels, therefore inheriting the nest including the worker bees and other resources. In a population there should also be simultaneous selective pressure on the queens to suppress this selfish and aggressive behavior, resulting in intragenomic conflict similar to the *Peromyscus* mouse example given earlier (Queller, 2003). This level of intracolonial aggression also needs to be balanced with the need for appropriate extra-colonial aggression (i.e., colony defense). The genomic conflict could occur through paternal piRNA silencing to increase aggression and maternal piRNA licensing to mitigate the paternal silencing and reduce aggression. Similar to the WTT and ELBOs discussed above this is a phenotype that would normally be suppressed (or canalized) but which would vary in extent between populations due to differing selective pressures (e.g., within AHB and EHB lineages; **Figure 4**).

The implication of our admittedly speculative model is that wider crosses in honeybees can result in increased aggression in one of the hybrid families because the cross disrupts the balance of genomic conflict for/against aggression. This could occur in any case where the extent of genomic conflict differs between lineages and might explain why beekeepers who cross different races of bees sometimes report higher aggression in one of the reciprocal families, though individual crosses would need to be investigated to gain a full understanding of this phenomenon (Adam, 1983). The selection for highly aggressive AHB colonies to use for our crosses might have resulted in the epigenetic release of this phenotype in that parent colony (i.e., simultaneously selected for increased paternal silencing of alleles that leads to aggression and less maternal opposition to this silencing), which explains the asymmetrically high aggression in the EA family (**Figure 4**). The importance of selecting for aggressive traits in the AHB parent may explain why another study that used EHB × AHB crosses similar to ours, but that didn’t select for differential aggression, did not have these same asymmetries in allele-specific expression (Galbraith and Grozinger, personal communication).

This piRNA mediated aggression model can be tested in multiple ways. Isolating and sequencing small RNAs from both sperm and eggs of the parent colonies would allow us to determine whether piRNAs are present and whether they target the genes that show biased allele usage. If they are present, the total RNA from sperm derived from drones of AHB colonies selected for high and low aggression could be injected into eggs from an EHB queen crossed with an AHB drone from a colony selected to be docile (the eggs must still be F1 hybrids to ensure both alleles are present for sequence specificity). Allele use can then be assessed in the resulting offspring, with the prediction that eggs injected with RNA derived from the “aggressive drones” will result in biased allele use in these offspring while those injected with RNA from the “docile drones” will not. Further experiments could then be performed to analyze brain metabolism and aggression in both sets of offspring. This model can also be tested through RNAi-mediated knockdown of the piRNA machinery in the parent EHB and AHB queens and drones (from colonies selected for high and low aggression) and subsequent testing of hybrid allele usage, metabolism, and aggression. Biased allele use and aggression would be expected to be lower when the machinery is knocked down paternally. Knockdowns could also be used in crosses between the highly aggressive AHB colonies. The cross of AHB^piRNA-^ queens × AHB^piRNA+^ drones would be expected to have high aggression (perhaps even higher than the parents) due to a loss of maternal licensing and AHB^piRNA+^ queen × AHB^piRNA-^ drones would be expected to have lower aggression (**Figure 4**).

The patterns that we observed in this family likely represent an inappropriate utilization of the retrograde signaling pathway because the maternal bias in gene expression in the EA family seems to occur in all tissues across life stages and not just in the brains as described for these metabolic changes in aggressive bees (Alaux et al., 2009; Li-Byarlay et al., 2014). Similarly, the metabolic deficiency in EA hybrids occurs at the whole body level even though aggressive bees are known to have increased oxidative metabolic rates at the whole body level in response to alarm pheromone (Moritz et al., 1985). Taken together, these results indicate that the whole organism-level asymmetric hybrid effects on allelic gene expression, metabolism, and aggression may be due to perturbations of established nuclear-mitochondrial signaling pathways that normally modulate brain metabolism and aggression in honeybees. While these results mark an important step in our understanding of aggression and describe a new pattern of hybrid gene regulation, additional work is necessary to better understand how differential allele expression acts on these traits, how this allelic expression is controlled and how these signaling pathways modulate aggression in the context of honeybee natural history.

## Author Contributions

GH and MA planned hybrid crosses, collected samples and performed stinging behavior assays. MA performed the crosses. JG, JT, and GH performed bioinformatics analyses. JG and GH wrote the manuscript with input from all authors.

## Conflict of Interest Statement

The authors declare that the research was conducted in the absence of any commercial or financial relationships that could be construed as a potential conflict of interest.
