# Supplementary material for: Biased Allele Expression and Aggression in Hybrid Honeybees may be Influenced by Inappropriate Nuclear-Cytoplasmic Signaling
Source: Front Genet. 2015 Dec 1;6:343. doi: 10.3389/fgene.2015.00343 (PMC4664729; doi:10.3389/fgene.2015.00343)
Supplement: Supplementary file 2 [file Supplementary_File_1.DOCX]

**Supplementary Tables**

**Supplementary Table 1:** Biased gene information.

A: Unique ID for each transcript in our analysis.

B: Best matching OGS3.2 gene ID, based on genomic location of gene and differentiating SNPs as well as BLAST results.

C-E: Location of the transcript on Amel4.5 chromosomes and scaffolds.

F: Annotation of transcripts, derived from *Apis mellifera* OGS3.2 annotations as well as BLAST comparison to other organisms.

G-I: Best matching *Drosophila* ortholog based on BLAST comparison.

J-L: Overlapping genes between our differential expression analysis and biased genes.

M-O: parent FDR values from Kocher et al 2015 (significant parent effect).

P-U: Maternal bias values and heat map of all biased genes.

V-BA: Sample types in each bias category that are significant at 60%, 70%, 80% and 90% bias cutoff values.

BB-BE: Genes that are biased in multiple categories across samples at 60%, 70%, 80% and 90% cutoff values.

BF-BI: Overlap with differentially expressed genes in aggressive and non-aggressive bees (Alaux et al 2009).

**Supplementary Table 2:** Gene counts of each category across samples and bias cutoff values.

A: Sample type

B-AG: Number of genes falling into each category at different bias cutoff values, broken down by sample type.

AH-AK: Number of genes falling into multiple categories using different bias cutoff values.

**Supplementary Table 3:** Differentially expressed gene information.

A-B: OGS3.2 and 1.1 gene IDs. OGS3.2 was used for mapping genes in the differential expression analysis.

C-D: Direction of expression difference in EA family.

E-S: Empirical analysis of DGE statistical results for each sample comparison.

T-V: Best matching *Drosophila* ortholog based on BLAST comparison.

W: Annotation of transcripts, derived from *Apis mellifera* OGS3.2 annotations as well as BLAST comparison to other organisms.

X-AA: Overlap with differentially expressed genes in aggressive and non-aggressive bees (Alaux et al 2009).

**Supplementary Table 4:** GO analysis results for European maternal only biased gene set based on best Drosophila orthologs.

A. GO term

B. GO ID

C. Number of times GO term is found in background Drosophila gene set.

D. Number of times GO term is found in European maternal only biased gene set.

E. Number of times GO term is expected in European maternal only biased gene set.

F. Bonferroni-corrected p-values for enrichment.

**Supplementary Figure 1**: Graph of total gene count in each bias category at different bias cutoff values. Bias calculated as maternal or paternal reads/total reads and represented as percentage of total reads

**Differential expression analysis**

The bees in this study were not cross-fostered in their hives (except those for brain samples) and so it is possible that differences in expression between the families could be largely due to differences between the colonies themselves and not due to the hybrid cross. The annotations of the bee genes seem to indicate that some of the genes are involved in either stress or immune responses, which could easily vary between colonies. Moreover, over 60% of the differentially expressed genes are found in guards, which are likely to be the sample with the most variation due to environmental sources in contrast to either instars or individual brains. This is because they are whole body samples and should represent all processes occurring in the bees, including physiological responses to the environment, as compared to brains, which are more restricted in the processes stemming from their gene expression. The instars were also whole body samples, but instar expression should be dominated by developmental expression patterns that shouldn’t vary between colonies. Further supporting this, there is no apparent connection between these differentially expressed genes and either our biased genes (only 13 genes overlap both lists) or the differentially expressed genes in aggressive versus non-aggressive bees of Alaux *et al.,* (2009). While roughly a quarter of our differentially expressed genes overlapped these “aggression-related” genes, there aren’t more than expected by chance in any category of bias and there is no pattern relating the expression of these between the two studies (Supplemental Table 2).

**Gene Descriptions** (including those discussed in manuscript):

Hexokinase 2 (HK2; GB47079) has been shown to be the key regulator of the metabolic shift between oxidative respiration and aerobic glycolysis in neuron progenitors (Gershon *et al.,* 2013). HK2 is induced by posphoinositide 3-kinase (Pi3k; GB42200), which is modulated by intracellular calcium levels through interactions with calmodulin (GB49410) and calcium/calmodulin-dependent protein kinase II (GB49535; Joyal et al 1997; Belgacem & Boradinsky, 2011). In addition to its role in the switch to aerobic glycolysis HK2 also binds with a voltage dependent anion channel (VDAC; GB49313) to inhibit apoptosis, though the mechanism underlying this is not completely understood (McCommis & Baines, 2012). One possible role is by HK2 altering VDAC structure to prevent B-cell lymphoma 2 (Bcl2) family proteins from binding with VDAC (McCommis & Baines, 2012). Notably, while some Bcl 2 proteins negatively regulate apoptosis, others may induce apoptosis (BNIP3; GB50136). Bag1 (GB54219) is a gene that has been shown to associate with Bcl-2 proteins and may enhance their effects on apoptosis (King *et al.,* 2001). Bag-1 is also a cochaperone with HOP (Hsp70/Hsp90 organizing protein; GB44056) and Hsp40 (GB54211) to regulate Hsp70 (not tested; NT) and Hsp90 (NT) interactions with P53 (NT), which can increase aerobic glycolysis by activating HK2 (Vousden & Ryan, 2009). Independent of this P53/HK2 interaction, Hsp40 has also been shown to regulate aerobic glycolysis by facilitating the degredation of another key regulator of aerobic glycolysis, pyruvate kinase isoform 2 (Pkm2; NT) through its interaction with Hsc70 (constitutively expressed form of Hsp70; GB49117; Huang *et al.,* 2014). In addition to activating HK2, P53 also activates AMP-activated protein kinase (AMPK; NT), a major regulator of cellular energy status, both directly and through its interactions with Sestrin (GB49567; Lee *et al.,* 2010). AMPK regulates ATP levels in the cell through interactions with nucleotide diphosphate kinase (NDPK; GB55139; Onyenwoke *et al.,* 2012). A recent study found that AMPK is also activated by overexpression of a mitochondrial uncoupling protein (UCP2; GB49259) and that this results in decreased expression of HK2 as well as reduced localization of the HK2 protein to the mitochondria (Esteves *et al.,* 2014). AMPK modulates the Target or Rapamycin (TOR) pathway, a key component linking cellular nutrition status with mitochondrial retrograde signaling (Jazwinski & Kriete, 2012). AMPK has been shown to colocalize with Aurora kinase (AurA; GB43732) during cell division and combined inhibition of both TOR and AurA has been shown to halt tumor cell growth, further connecting these signaling pathways (Vazquez-Martin *et al.,* 2014; Savannah *et al.,* 2012). The TOR pathway is also regulated by Pi3k signaling in response to insulin receptors (GB49397) and insulin like growth factors (GB53353; Lee *et al.,* 2010). Taken together, these various genes relating to metabolic shifts indicate that the bias in our gene set may be due to mitochondrial retrograde signaling.

In addition to their roles in metabolic shifts, some of the genes/pathways discussed above also play a role in changes in gene transcription and translation, which may help to elucidate the mechanism underlying this allelic bias. TOR has been shown to regulate RNA polymerase III to control growth in *Drosophila*, and our gene set includes two subunits of this protein complex (GB41332 and GB49307; Marshall *et al.,* 2012). TOR signaling also influences circadian rhythms through S6 kinase (S6k; GB46786) and interacts with glycogen synthase kinase 3 (GSK3; shaggy GB53394: Zheng & Sehgal, 2010). GSK3 itself regulates many transcription factors and has also been shown to affect the accessibility of chromatin (Park *et al.,* 2011). Our gene set also has several genes that are part of the piwi-interacting RNA (piRNA) pathway. piRNAs are small noncoding RNAs that are highly enriched in the germline where they silence transposons and act on epigenetic regulation in a sequence-specific manner in somatic cells by modifying chromatin through recruitment of Heterochromatin Protein I (NT) and polycomb/trithorax-group proteins (CG11970, GB43163; Brahma, GB52563; Kismet, GB51962; Trithorax-related, GB41196; Kal *et al.,* 2000; Daubresse *et al.,* 1999; Peng & Lin, 2013; Huang *et al.,* 2013; Srinivasan *et al.,* 2005). piRNA biogenesis occurs in nuages, granules that are localized near the mitochondria, and this close association is apparent in the necessity of Minotaur (GB50105), a mitochondrial membrane protein, in piRNA biogenesis (Shiromoto *et al.,* 2013). Some genes that are involved in metabolism are also related to this pathway. HOP, discussed above in relation to metabolic shifts, regulates the piRNA pathway through interactions with Hsp90 (Vamsi *et al.,* 2011). Tor has also been shown to be regulated by piRNAs in the germline of *C. elegans* (Barberan-Soler *et al*., 2014). Another gene, Papi (GB49404), complexes with Piwi proteins to recruit them to the nuage to assemble the piRNA pathway components (Liu *et al*., 2011). A gene that we could not test but that lies in the middle of one of our highly biased gene clusters, Pimet (GB49074), is important for processing piRNAs (Saito et al 2007).

The genes described above clearly have multiple and diverse functions and much future work will be required to elucidate what role they may play in the asymmetric PSGE and other phenotypic asymmetries that we observe in these hybrids honeybees.

**Supplemental references:**

Alaux, C., Sinha, S., Hasadsri, L., Hunt, G.J., Guzman-Novoa, E., Degrandi-Hoffman, G., et al. (2009). Honey bee aggression supports a link between gene regulation and behavioral evolution. Proc Natl Acad Sci USA 106, 15400-15405. doi: 10.1073/Pnas.0907043106.

Barberan-Soler, S., Fontrodona, L., Ribo, A., Lamm, A.T., Iannone, C., Ceron, J., et al. (2014). Co-option of the piRNA Pathway for Germline-Specific Alternative Splicing of C-elegans TOR. Cell Rep 8, 1609-1616. doi: 10.1016/J.Celrep.2014.08.016.

Belgacem, Y.H., and Borodinsky, L.N. (2011). Sonic hedgehog signaling is decoded by calcium spike activity in the developing spinal cord. Proc Natl Acad Sci USA 108, 4482-4487. doi: 10.1073/Pnas.1018217108.

Daubresse, G., Deuring, R., Moore, L., Papoulas, O., Zakrajsek, I., Waldrip, W.R., et al. (1999). The Drosophila kismet gene is related to chromatin-remodeling factors and is required for both segmentation and segment identity. Development 126, 1175-1187.

Esteves, P., Pecqueur, C., Ransy, C., Esnous, C., Lenoir, V., Bouillaud, F., et al. (2014). Mitochondrial Retrograde Signaling Mediated by UCP2 Inhibits Cancer Cell Proliferation and Tumorigenesis. Cancer Res 74, 3971-3982. doi: 10.1158/0008-5472.Can-13-3383.

Gershon, T.R., Crowther, A.J., Tikunov, A., Garcia, I., Annis, R., Yuan, H., et al. (2013). Hexokinase-2-mediated aerobic glycolysis is integral to cerebellar neurogenesis and pathogenesis of medulloblastoma. Cancer and Metabolism 1.

Huang, X.A., Yin, H., Sweeney, S., Raha, D., Snyder, M., and Lin, H. (2013). A major epigenetic programming mechanism guided by piRNAs. Developmental Cell 24, 502-516. doi: 10.1016/j.devcel.2013.01.023.

Huang, L.Q., Yu, Z.H., Zhang, T., Zhao, X.P., and Huang, G. (2014). HSP40 Interacts with Pyruvate Kinase M2 and Regulates Glycolysis and Cell Proliferation in Tumor Cells. PLoS ONE 9. doi: 10.1371/journal.pone.0092949.

Jazwinski, S.M., and Kriete, A. (2012). The yeast retrograde response as a model of intracellular signaling of rnitochondrial dysfunction. Front Physiol 3. doi: 10.3389/Fphys.2012.00139.

Joyal, J.L., Burks, D.J., Pons, S., Matter, W.F., Vlahos, C.J., White, M.F., et al. (1997). Communication - Calmodulin activates phosphatidylinositol 3-kinase. J Biol Chem 272, 28183-28186. doi: 10.1074/Jbc.272.45.28183.

Kal, A.J., Mahmoudi, T., Zak, N.B., and Verrijzer, C.P. (2000). The Drosophila Brahma complex is an essential coactivator for the trithorax group protein Zeste. Gene Dev 14, 1058-1071.

King, F.W., Wawrzynow, A., Hohfeld, J., and Zylicz, M. (2001). Co-chaperones Bag-1, Hop and Hsp40 regulate Hsc70 and Hsp9O interactions with wild-type or mutant p53. Embo J 20, 6297-6305. doi: Doi 10.1093/Emboj/20.22.6297.

Lee, J.H., Budanov, A.V., Park, E.J., Birse, R., Kim, T.E., Perkins, G.A., et al. (2010). Sestrin as a Feedback Inhibitor of TOR That Prevents Age-Related Pathologies. Science 327, 1223-1228. doi: 10.1126/Science.1182228.

Liu, L., Qi, H., Wang, J., and Lin, H. (2011). PAPI, a novel TUDOR-domain protein, complexes with AGO3, ME31B and TRAL in the nuage to silence transposition. Development 138, 1863-1873. doi: 10.1242/dev.059287.

Marshall, L., Rideout, E.J., and Grewal, S.S. (2012). Nutrient/TOR-dependent regulation of RNA polymerase III controls tissue and organismal growth in Drosophila. Embo J 31, 1916-1930. doi: 10.1038/Emboj.2012.33.

McCommis, K.S., and Baines, C.P. (2012). The role of VDAC in cell death: Friend or foe? Bba-Biomembranes 1818, 1444-1450. doi: 10.1016/j.bbamem.2011.10.025.

Onyenwoke, R.U., Forsberg, L.J., Liu, L., Williams, T., Alzate, O., and Brenman, J.E. (2012). AMPK directly inhibits NDPK through a phosphoserine switch to maintain cellular homeostasis. Mol Biol Cell 23, 381-389. doi: 10.1091/mbc.E11-08-0699.

Park, S.H., Park-Min, K.H., Chen, J., Hu, X.Y., and Ivashkiv, L.B. (2011). Tumor necrosis factor induces GSK3 kinase-mediated cross-tolerance to endotoxin in macrophages. Nat Immunol 12, 607-U158. doi: 10.1038/Ni.2043.

Peng, J.C., and Lin, H. (2013). Beyond transposons: the epigenetic and somatic functions of the Piwi-piRNA mechanism. Curr Opin Cell Biol 25, 190-194. doi: 10.1016/j.ceb.2013.01.010.

Saito, K., Sakaguchi, Y., Suzuki, T., Suzuki, T., Siomi, H., Siomi, M.C. (2007) Pimet, the Drosophila homolog of HEN1, mediates 2 '-O-methylation of PIWI-interacting RNAs at their 3 ' ends. Genes and Development 21, 1603-1608. doi: 10.1101/gad.1563607

Savannah, K.J.B., Demicco, E.G., Lusby, K., Ghadimi, M.P.H., Belousov, R., Young, E., et al. (2012). Dual Targeting of mTOR and Aurora-A Kinase for the Treatment of Uterine Leiomyosarcoma. Clin Cancer Res 18, 4633-4645. doi: 10.1158/1078-0432.Ccr-12-0436.

Shiromoto, Y., Kuramochi-Miyagawa, S., Daiba, A., Chuma, S., Katanaya, A., Katsumata, A., et al. (2013). GPAT2, a mitochondrial outer membrane protein, in piRNA biogenesis in germline stem cells. Rna-a Publication of the Rna Society 19, 803-810. doi: 10.1261/Rna.038521.113.

Srinivasan, S., Armstrong, J.A., Deuring, R., Dahlsveen, I.K., McNeill, H., Tamkun, J.W. (2005) The Drosophila trithorax group protein Kismet facilitates an early step in transcriptional elongation by RNA Polymerase II. Development 132, 1623-1635. doi: 10.1242/dev.01713

Vazquez-Martin, A., Oliveras-Ferraros, C., and Menendez, J.A. (2014). The active form of the metabolic sensor AMP-activated protein kinase α (AMPKα) directly binds the mitotic apparatus and travels from centrosomes to the spindle midzone during mitosis and cytokinesis. Cell Cycle 8, 2385-2398. doi: 10.4161/cc.8.15.9082.

Vousden, K.H., and Ryan, K.M. (2009). p53 and metabolism. Nat Rev Cancer 9, 691-700. doi: 10.1038/nrc2715.

Zheng, X., and Sehgal, A. (2010). AKT and TOR signaling set the pace of the circadian pacemaker. Curr Biol 20, 1203-1208. doi: 10.1016/j.cub.2010.05.027.
